# On the Medical Use of Phosphorus

**Published:** 1800-08

**Authors:** 


### *On the Medical Ufe of Phofphorus.*

PHOSPHORUS, whofe ufe was introduced into medicine about fifty years ago, is known as the moft powerful exciting remedy which we poffefs. Several phyficians have tried it, with more or lefs fuccefs, in all cafes where a high degree of nervous debility prevails; and accordingly, in nervous fevers, palfies, impotentia virilis, epilepfies, &c. alfo in arthritis nodofa et atonica, it has proved very efficacious; and it likewife deferves to be ufed in cafes of perfons poifoned with lead or arfenic. There exifts, at leaft, an inftance of a perfon, who having been given poifon in Italy, (probably aqua toffana,) became quite emaciated, his nails and hair fell off fpontaneoufly, and his life was only faved by phofphorous. Its effects are at the fame time in a confiderable degree diuretic and diaphoretic.

Experience has, however, fhown that the application of this remedy requires the utmoft caution, and it may fometimes prove very dangerous if proper care is not taken; as it has been obferved, either to caufe a vehement inflammation in the ftomach and bowels, accompanied with very burning pains in the regio hypochondriaca, and often terminating in death, or by producing indurations, or fcirrhus of the ftomach, which occafion chronic cardialgies, indigeftions, an habitual vomition of whatever is taken, emaciation, and hectic death. Thefe ill confequences confequences are in a great meafure owing to the mode in which it is adminiftered; either by giving it in fubftance, or in too great a dofe; as, in the firft cafe particularly, the minuteft particle of it may, by adhering to the ftomach, produce an inflammation, or at leaft an induration.

As for the dofe, it has been obferved, that it never muft exceed two grains in 24 hours, and a burning pain or fenfation was always perceived whenever it was given in a greater quantity, but generally one grain in 24 hours will be fufficient to produce the defired effects.

The form in which it is to be exhibited ought to poffefs the quality of containing the phofphorus quite diffolved, and at the fame time of obtunding its too dangerous irritation upon the ftomach. The forms hitherto ufed were a mixture with a conferve, which is of all others the moft improper, becaufe the remedy is applied here in fubftance, and may of courfe eafily bring on the above mentioned fymptoms; a folution of it in oil, which, though otherwife very proper, is too difagreeable to take; a folution in ether, or naphta vitrioli, in which the phofphorus entirely diffolves; but, by this combination, its heating and irritating qualities are exceedingly increafed; and as only eight grains of phofphorus are foluble in one ounce of the ether, 120 drops muft be taken, only to bring one grain of it into the body, which in moft cafes would be too dangerous. However, this naphta phofphorata may be of great benefit or ufe in a very high degree of nervous debility, combined with a total want of fenfation.

The following form of giving phofphorus internally, will therefore be the moft proper, on account of containing it diffolved and equally divided throughout, and having it fufficiently involved in mucilage, to prevent any ill confequences from a local irritation of the ftomach even in very fenfible conftitutions. The phofphorus is rubbed with fweet almonds, or a fufficient quantity of gum arabic, to make an emulfion, and by an addition of fpiritus nitri dulcis, or liquor anodynus, the tafte and fmell of the phofphorus is rendered lefs difagreeable.—

℞. *Phofphor. urinæ gr. ii. fubigantur longa trituratione cum mucilagine gr. arabici q. s. ut fiat cum aquæ fontan unc. vj. emulfio, cui adde fyrupe de althaea unc. j. liquor. anodyn. miner. Hoffmann. gutt. xxx.* D. S. *omni bihorio cochlear fumendum aut plus. pro re nata.* In this way a very efficacious folution will be obtained, which fhines in the dark, and is agreeable enough to be taken. *(From Hufeland's Practicul Journal, vol.* vii. *No.* 3, 1799.*)* Of the external ufe of phofphorus, particularly in caries offium, we fhall, perhaps, fay fomething in one of the future numbers of this Journal.

CRITICAL

